# Analysis of Genomic Copy Number Variation in Miscarriages During Early and Middle Pregnancy

**DOI:** 10.3389/fgene.2021.732419

**Published:** 2021-09-17

**Authors:** Heming Wu, Qingyan Huang, Xia Zhang, Zhikang Yu, Zhixiong Zhong

**Affiliations:** ^1^ Center for Precision Medicine, Meizhou People’s Hospital (Huangtang Hospital), Meizhou Academy of Medical Sciences, Meizhou, China; ^2^ Guangdong Provincial Key Laboratory of Precision Medicine and Clinical Translational Research of Hakka Population, Meizhou People’s Hospital (Huangtang Hospital), Meizhou Academy of Medical Sciences, Meizhou, China; ^3^ Center for Prenatal Diagnosis, Meizhou People’s Hospital (Huangtang Hospital), Meizhou Academy of Medical Sciences, Meizhou, China

**Keywords:** copy number variation, miscarriage, early pregnancy, middle pregnancy, copy number variation sequencing (CNV-seq)

## Abstract

The purpose of this study was to explore the copy number variations (CNVs) associated with miscarriage during early and middle pregnancy and provide useful genetic guidance for pregnancy and prenatal diagnosis. A total of 505 fetal specimens were collected and CNV sequencing (CNV-seq) analysis was performed to determine the types and clinical significance of CNVs, and relevant medical records were collected. The chromosomal abnormality rate was 54.3% (274/505), among which the numerical chromosomal abnormality rate was 40.0% (202/505) and structural chromosomal abnormality rate was 14.3% (72/505). Chromosomal monosomy mainly occurred on sex chromosomes, and chromosomal trisomy mainly occurred on chromosomes 16, 22, 21, 15, 13, and 9. The incidence of numerical chromosomal abnormalities in ≥35 year-old age pregnant women was significantly higher than <35 year-old age group. The highest incidence of pathogenic CNV (pCNV) was found in fetuses at ≤6 weeks of pregnancy (5.26%), and the incidence of variants of unknown significance (VOUS) CNVs decreased gradually with the increase of gestational age. The rate of chromosomal abnormalities of fetuses in early pregnancy (59.5%) was higher than that of fetuses in middle pregnancy (27.2%) (*p* < 0.001). There were 168 genes in VOUS + pCNV regions. 41 functions and 12 pathways (*p* < 0.05) were enriched of these genes by Gene Ontology (GO) analysis and Kyoto Encyclopedia of Genes and Genomes (KEGG) analysis. Some meaningful genetic etiology information such as genes and pathways has been obtained, it may provide useful genetic guidance for pregnancy and prenatal diagnosis.

## Introduction

Miscarriage is a clinical event that termination of pregnancy at less than 28 weeks, and the fetal weight is less than 1000 g, occurs in before 12 gestational weeks is called miscarriage during early pregnancy, occurs in 13–27 gestational weeks is called miscarriage during middle pregnancy (Muttukrishna et al., 2002). The incidence of miscarriage is about 15–20%, and there is a trend of increasing year by year (Quenby et al., 2021). The etiology of miscarriage is closely related to environmental factors (Zhou et al., 2017), genetic factors(Pereza et al., 2017), body immune state (Wang et al., 2021).

Studies have shown that genetic factors play an important role in early miscarriage, with about 50% of the cases caused by chromosomal abnormalities (van den Berg et al., 2012; Ozawa et al., 2019), while the risk factors of late miscarriage (≥28 gestational weeks) are mainly immune and environmental factors (Meng et al., 2020). Types of chromosomal abnormalities including numerical chromosomal abnormality, structural chromosomal abnormality, chimera, and polyploidy. In miscarriage cases, numerical chromosomal abnormality has the highest frequency (up to 90%), structural abnormality accounts for about 6%, and chimeras account for about 12% (Pérez-Durán et al., 2015; Zhang et al., 2019). A study has shown that copy number variations (CNVs) (known as microdeletion/microduplication) accounts for about 2.7% miscarriages, second only to aneuploidy and polyploidy (Wang Y. et al., 2020). CNVs are copy number changes of the genome, which variations can range in size from several dozens of bases (>50 bp) to megabases (Zarrei et al., 2015; Lauer and Gresham, 2019). Regardless of the cause, the etiological analysis of miscarriage is of great significance to the aborted fetuses and the next pregnancy of women with childbearing age.

Low-coverage massively parallel CNV-seq is a method for sequencing analysis of samples, and the sequencing results were compared with the human reference genome, and CNV was found through bioinformatics analysis (Liang et al., 2014). It is a high-resolution and low-cost technology for detecting CNVs in clinical samples. It can detect chromosome aneuploidies, polyploidies, and CNVs, microdeletions and microduplications with a chromosome resolution of 0.1 Mb (Xie and Tammi, 2009; Liang et al., 2014; Dong et al., 2016). In addition, it can detect unknown variations, to find new genetic information related to the disease.

In the current study, chromosomal abnormalities of miscarriages during early and middle pregnancy were evaluated systematically. We further analyzed the genomic regions of detected chromosomal abnormalities to identify potential miscarriage-associated CNVs. Moreover, potential miscarriage candidate genes and gene functions were identified by gene enrichment analysis. This study is expected to provide meaningful data for the genetic etiology of aborted fetuses and for the next pregnancy of women with childbearing age.

## Materials and Methods

### Participants

Fetal samples of miscarriages during early and middle pregnancy were collected from the Department of Obstetrics, Meizhou People’s Hospital, China, from 2017 to 2020. All parents consented to test voluntarily and provided signed informed consent. The gestational age at the time of miscarriage ranges from 4 to 27 weeks. These fetuses were in the early and middle stages of pregnancy. The study was performed under the guidance of the Declaration of Helsinki and approved by the Ethics Committee of Meizhou People’s Hospital (Clearance No.: 2016-A-45).

### CNV-seq Detection and Data Analysis

Approximately 5–10 mg of fetal tissue was selected under the microscope, minced into pieces, and rinsed with sterile physiological saline. Genomic DNAs were extracted using DNAeasy Kit (Qiagen, Valencia, CA, United States). DNA quality was evaluated using a NanoDrop™ spectrophotometer (Thermo Fisher Scientific, Inc.). Genomic DNAs were fragmented, then were endligated with barcoded sequence adaptors. Tagged DNA fragments were amplified using primers corresponding to adaptor sequences to generate sequencing libraries. Finally, sequencing was performed on BioelectronSeq 4000 Platform (semiconductor sequencing system) (Thermo Fisher) at an approximately 1× depth. After the sequencing is completed, the obtained fastq data is filtered by bioinformatics software. Burrows-Wheeler algorithm was applied to calculate the change of copy number of each sequencing sequence, taking HG19 genome sequence as reference (window size was 10 kb), to obtain the copy number value of each chromosome, determine the duplication or deletion of chromosome fragments, and finally draw the detection results map. Algorithms for calculating CNV include: 1) Determine the CNV value of adjusted data according to Circular Binary Segmentation algorithm. 2) CNV value was determined by Hidden Markov Model algorithm. 3) The significance of CNV within the interval was further analyzed according to Z-score. The defined reference range is: the reference range of R value is between −0.2 and 0.2, and the reference range of Z value is between −3 and 3. If R value >0.2 or <−0.2, and Z value >3 or <−3, it indicates the presence of duplication or deletion in the chromosomal region.

The tested data was analyzed according to Database of Genomic Variants (DGV) (http://dgv.tcag.ca/dgv/app/homr), Database of Genomic Variation and Phenotype in Humans using Ensembl Resources (DECIPHER) (http://decipher.sanger.ac.uk), Online Mendelian Inheritance in Man (OMIM) database (http://www.omim.org), and the University of California Santa Cruz Database (UCSC) (https://genome.ucsc.edu). According to the American College of Medical Genetics and Genomics (ACMG) guidelines, the clinical significance of CNVs is divided into 5 grades: pathogenic CNVs (pCNVs), likely pathogenic CNVs, variants of uncertain significance (VOUS) CNVs, likely benign CNVs, and benign CNVs (Brandt et al., 2020; Riggs et al., 2020).

### Statistical Analysis

SPSS statistical software version 21.0 was used for data analysis. Data was reported with the descriptive statistics method and measurement data was expressed as mean ± standard deviation (SD). Chi-square test was used to analyze the difference among the groups. A value of *p* < 0.05 was considered as statistically significant.

### Functional Enrichment Analysis

The genes located in the pathogenic CNVs, likely pathogenic CNVs and VOUS regions were referred to in the DECIPHER database (http://decipher.sanger.ac.uk). Enrichment analysis was tested for the functional categories defined in Gene Ontology (GO) and Kyoto Encyclopedia of Genes and Genomes (KEGG) by clusterProfiler package in R 3.6.3. In the current study, *p* < 0.05 was considered as statistically significant enrichment.

## Results

### Baseline Characteristics of Pregnant Women and Overall CNV Results of Fetuses

A total of 505 fetal tissue samples were collected for CNV-seq in this study. The average age of the pregnant women was 29.98 ± 5.07 years, and the average gestational duration of fetuses was 10.28 ± 4.33 weeks. There were 261 (51.7%) pregnant women under the age of 30 years, 142 (28.1%) pregnant women between 30 and 34 years of age, 78 (15.4%) pregnant women between 35 and 39 years of age, and 24 (4.8%) pregnant women over 40 years of age. Among these fetuses with miscarriage, there were 410 (81.2%) fetuses aborted in the early pregnancy (≤12 weeks) and 95 (18.8%) fetuses in the middle pregnancy (13–27 weeks). Chromosomal abnormalities were found in 274 fetuses and no chromosomal abnormalities were found in 231 fetuses. The chromosomal abnormality rate was 54.3% (274/505), and the normal rate was 45.7% (231/505). There were 202 cases (40.0%) had numerical chromosomal abnormalities, including 150 cases (29.7%) with autosomal trisomy, 24 cases (4.8%) with sex-chromosome monosomy, 3 cases (0.6%) with autosomal monosomy, 1 case (0.2%) with sex-chromosome trisomy, and 1 case (0.2%) with autosomal tetrasomy. There were 72 cases (14.3%) had structural chromosomal abnormalities, including 48 cases (9.5%) with VOUS CNVs, 11 cases (2.2%) with pCNVs, 3 cases (0.6%) with likely pCNVs, and 6 cases (1.2%) with benign CNVs. Among VOUS CNVs, pCNVs, and likely pCNVs, 51 (56.0%) with <1 Mb CNVs, 17 (18.7%) with 1–10 Mb CNVs, and 23 (25.3%) with >10 Mb CNVs (Table 1).

**TABLE 1:** Demographic variables and baseline characteristics of mothers who suffer from miscarriages and overall CNV results of fetuses.

Characteristics	Number	Proportion/constituent ratio (%)
Age of mothers who had miscarriages (29.98 ± 5.07 years)		
<30	261	51.7
30–34	142	28.1
35–39	78	15.4
≥40	24	4.8
Gestational week of fetuses (10.28 ± 4.33 W)		
Early pregnancy (≤12 W)	410	81.2
Middle pregnancy (13–27 W)	95	18.8
CNVs result		
Chromosomal number abnormality	202	40.0
Autosomal trisomy	150	29.7
Sex chromosome monosomy	24	4.8
Autosomal monosomy	3	0.6
Sex chromosome trisomy	1	0.2
Autosomal tetrasomy	1	0.2
Chimera	23	4.6
Chromosomal structural abnormality	72	14.3
VOUS CNV	48	9.5
pCNV	11	2.2
Likely pCNV	3	0.6
Benign CNV	6	1.2
pCNV + likely pCNV	1	0.2
pCNV + VOUS CNV	1	0.2
likely pCNV + VOUS CNV	2	0.4
Normal	231	45.7
Size distribution of the VOUS CNVs, pCNVs and likely pCNVs		
<1 Mb	51	56.0
1–10 Mb	17	18.7
>10 Mb	23	25.3

CNV, copy number variation; y, years; w, weeks; VOUS, variants of uncertain significance; pCNV, pathogenic CNV; Mb, megabase.

### Results of Chromosomal Number Abnormalities, VOUS CNVs and pCNVs

**FIGURE 1:**
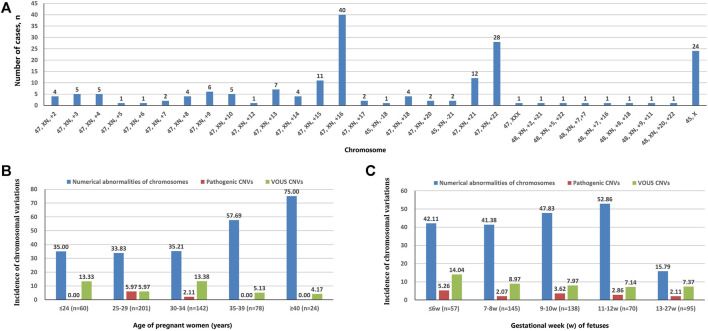
Chromosomal variation analysis. **(A)** The number of cases of chromosome aneuploidies (XN = XX or XY). **(B)** Incidence of numerical abnormalities of chromosome, pathogenic CNVs, and VOUS CNVs depending on age of pregnant women. **(C)** Chromosomal variations in miscarriage samples of different gestational weeks.

A total of 202 cases with numerical chromosomal abnormalities, including 173 cases with single-chromosome aneuploidies, 6 cases with two chromosomes aneuploidies, and 23 cases with chromosomal aneuploid chimera. In single-chromosome aneuploidies (chimeras were not counted), 47,XN,+16 (*n* = 40); 47,XN,+22 (*n* = 28); 45,X (*n* = 24); 47,XN,+21 (*n* = 12); 47,XN,+15 (*n* = 11); and 47,XN,+13 (*n* = 7) were the most common. In addition, one fetus with chromosome 7 tetrasomy (48,XN,+7,+7) was found (Figure 1A). The chromosomal aneuploid chimera karyotypes in 23 cases were 45,XN,−4[40%]/46,XN[60%]; 45,XN,−4[50%]/46,XN[50%]; 47,XN,+12[30%]/46,XN[70%]; 45,X[10%]/46,XN[90%]; 45,X[15%]/46,XN[85%]; 45,X[20%]/46,XN[80%]; 45,X[25%]/46,XN[75%]; 45,X[90%]/46,XN[10%]; 47,XN,+16[50%]/46,XN[50%]; 45,XN,-21[50%]/46,XN[50%]; 47,XN,+17[40%]/46,XN[60%]; 47,XN,+2[10%]/47,XN,+16[90%]; 47,XN,+2[15%]/46,XN[85%]; 47,XN,+2[60%]/46,XN[40%]; 47,XN,+13[40%]/47,XN,+21[40%]/47,XN,+22[20%]; 47,XN,+3[10%]/47,XN,+12[90%]; 45,X[15%]/46,XN[85%]; 45,X[25%]/46,XN[75%]; 47,XN,+12[15%]/46,XN[85%]; 47,XX[10%]/46,XN[90%]; 45,X[15%]/47,XN,+17[50%]/46,XN[35%]; and 47,XN,+4[15%]/47,XN,+18[15%]/46,XN[70%].

The incidence of numerical chromosomal abnormalities in ≥35 year-old age pregnant women was significantly higher than <35 year-old age group, and it increased continuously in pregnant women older than 25 years of age (from 33.83 to 75.00%). The pCNVs of fetuses mainly occurred in the 25–29 years old, and 30–34 years old pregnant women. There was no fetal pCNVs was detected in other age groups. The incidences of VOUS CNVs were not significantly different between these age groups (Figure 1B). The highest rate of numerical chromosomal abnormalities was found in miscarriage fetuses from 11 to 12 weeks of gestation (52.86%), followed by 9–10 weeks (47.83%), ≤6 weeks (42.11%), 7–8 weeks (41.38%), and 13–27 weeks (15.79%) of gestation. The highest incidence of pCNVs was found in ≤6 weeks of gestation group (5.26%), the incidences of pCNVs were not significantly different between gestational age groups. The incidences of VOUS CNVs decreased gradually with the increase of gestational age. (Figure 1C).

VOUS CNVs were detected in 48 fetal tissues, pCNVs and likely pCNVs were detected in 18 fetal tissues. The size of VOUS CNVs and pCNVs, as well as the location of the fragments in the genome, the genes contained in the fragment, and the related clinical diseases are shown in Supplemental Table 1.

### Identification of Candidate Genes for Miscarriages in Early and Middle Pregnancy

**FIGURE 2:**
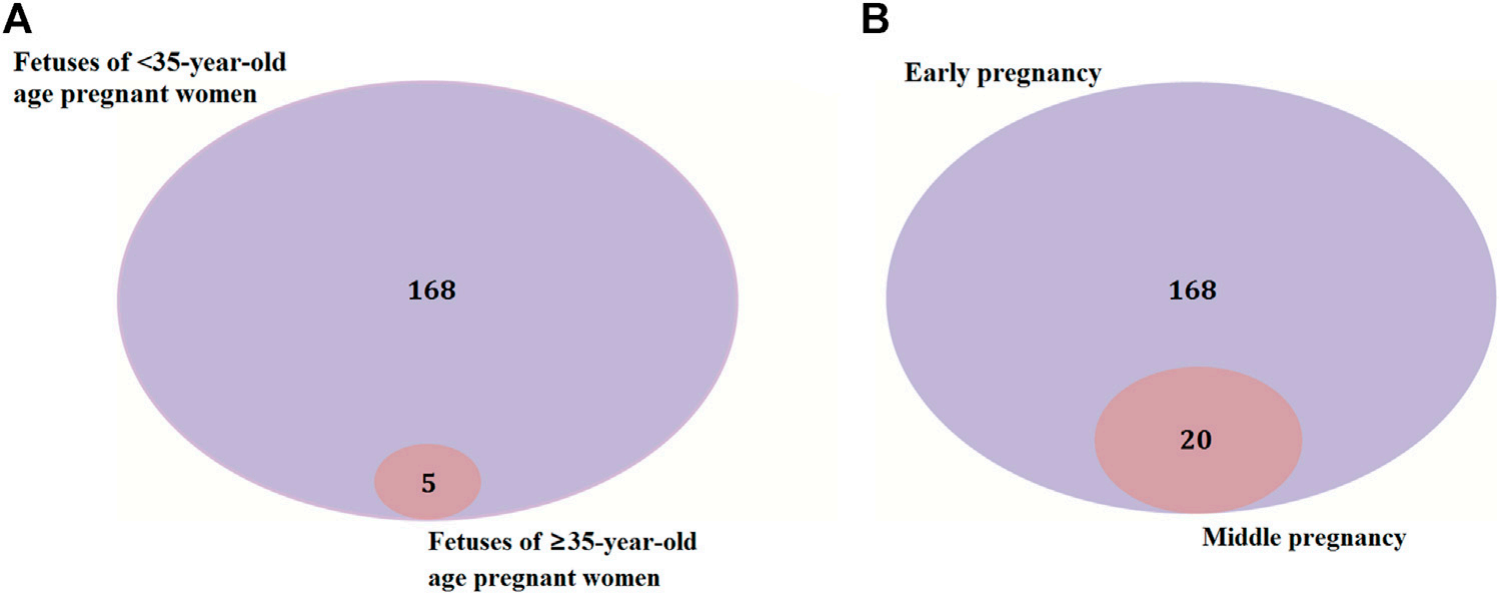
Venn diagrams of genes in VOUS + pathogenic CNV regions depending on age of pregnant women **(A)** and different pregnancies **(B)**.

**FIGURE 3:**
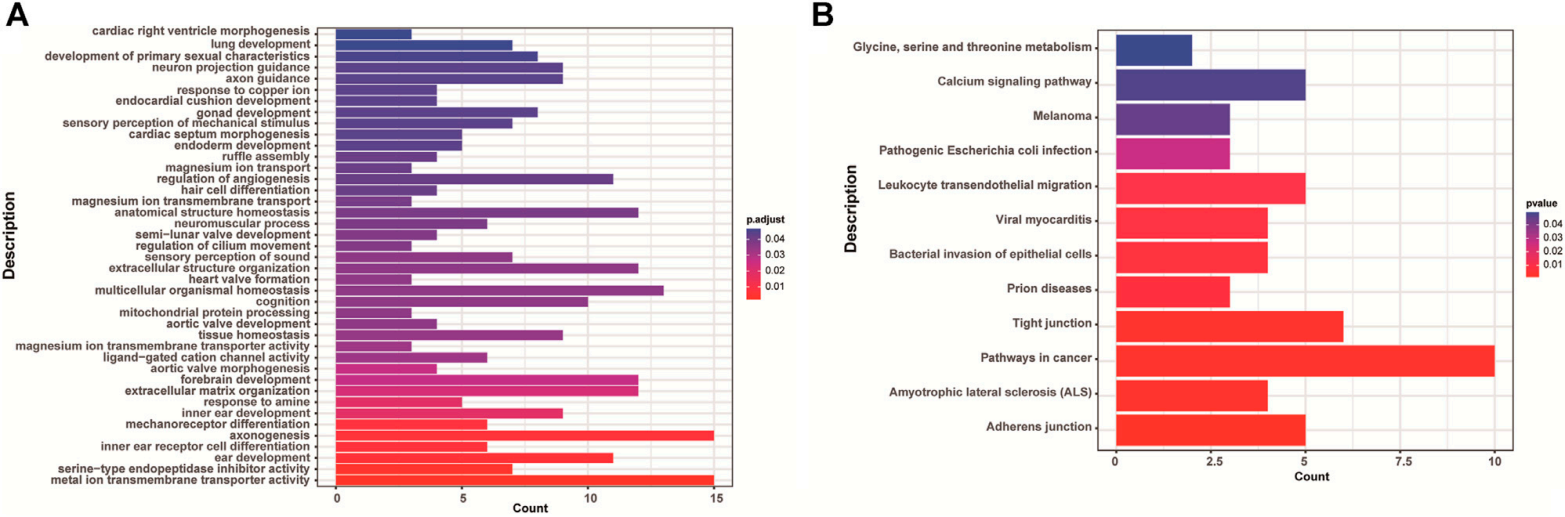
Enriched functions **(A)** and pathways **(B)** with adjust *p* < 0.05 of GO analysis and KEGG analysis.

There were 168 and five genes in VOUS CNVs + pCNVs + likely pCNVs in fetuses of <35 year-old age pregnant women and fetuses of ≥35 year-old age pregnant women, respectively. And there were 168 and 20 genes in VOUS CNVs + pCNVs + likely pCNVs in miscarriage fetuses in early pregnancy and middle pregnancy, respectively. The details are shown in Venn diagrams of number of genes in each group (Figure 2). To identify the critical genes and related signaling pathways associated with early and middle miscarriage, the genes in the VOUS CNVs + pCNVs + likely pCNVs regions were examined by the Gene Ontology (GO) analysis, Kyoto Encyclopedia of Genes and Genomes (KEGG) analysis. GO analysis showed that the 168 genes were significantly enriched in 41 different functions (*p* < 0.05), including 37 GO biological process (BP) terms, and four GO molecular function (MF) terms. The most significant of which was “metal ion transmembrane transporter activity” (*p* = 0.002), followed by “serine-type endopeptidase inhibitor activity” (*p* = 0.003), “ear development” (*p* = 0.006) and “inner ear receptor cell differentiation” (*p* = 0.007). Some biological processes were enriched such as organ differentiation and development, nervous system formation and development, transmembrane transport, and cellular functions (Figure 3A). KEGG analysis results showed that the most enriched pathways of these genes were adherens junction, amyotrophic lateral sclerosis, pathways in cancer, tight junction, and so on (Figure 3B).

### Comparison of CNVs Results Among Different Age Pregnant Women and Gestational Week of Fetuses

The subjects with both numerical chromosomal abnormality and structural chromosomal abnormality, as well as subjects with both VOUS CNV and pCNV were excluded. The rate of chromosomal abnormality was 50.1% (198/395) and 66.7% (68/102) in fetuses of <35 year-old age pregnant women and fetuses of ≥35 year-old age pregnant women, respectively. The difference was statistically significant (*p* = 0.004). In subjects with chromosomal abnormality, the rate of numerical chromosomal abnormalities in fetuses of ≥35 year-old age pregnant women (92.6%) was higher than that in fetuses of <35-year-old age pregnant women (70.2%), while the rate of structural chromosomal abnormalities in fetuses of ≥35 year-old age pregnant women (7.4%) was lower than that in fetuses of <35 year-old age pregnant women (29.8%) (*p* < 0.001) (Table 2).

**TABLE 2:** Comparison of CNVs results among different age pregnant women and gestational week of fetuses.

CNVs result	Average age of pregnant women				Gestational week of fetuses			
	<35 years (n, %)	≥35 years (n, %)	χ^2^	*p*	Early pregnancy (≤12 W) (n, %)	Middle pregnancy (13–27 W) (n, %)	χ^2^	*p*
Normal	197(49.9)	34(33.3)	8.915	0.004	164(40.5)	67(72.8)	31.505	<0.001
Chromosomal abnormalities	198(50.1)	68(66.7)			241(59.5)	25(27.2)		
Chromosome number abnormalities	139(70.2)	63(92.6)	13.956	<0.001	187(77.6)	15(60.0)	3.837	0.082
Chromosome structural abnormalities	59(29.8)	5(7.4)			54(22.4)	10(40.0)		
VOUS	39(66.1)	5(100)	1.497	0.574	37(68.5)	7(70.0)	0.442	0.672
pCNV + likely pCNV	12(20.3)	0(0)			11(20.4)	1(10.0)		

VOUS, variants of uncertain significance. y, years; w, weeks.

The rate of chromosomal abnormalities of fetuses in early pregnancy (59.5%) was higher than that of fetuses in middle pregnancy (27.2%) (*p* < 0.001), while there were no statistically differences in the incidences of structural chromosomal abnormalities and pCNVs between the early and middle pregnancy groups (all *p* > 0.05) (Table 2).

## Discussion

The causes of spontaneous miscarriage include endocrine factors (Arredondo and Noble, 2006), reproductive tract malformation (Venetis et al., 2014), infectious factors (Leisher et al., 2021), immune factors (Muyayalo et al., 2018), alcohol exposure (Sundermann et al., 2021), genetic factors (Lan et al., 2021) and some other factors that have not been clarified yet. Despite continuous medical advances, spontaneous abortion during early and middle pregnancy is still an important health problem. Many couples may face the risk of miscarriage when they choose to have children. It places a heavy psychological and financial burden on many families. Genetic factors are one of the main causes, fetal chromosomal abnormality is an important genetic factor of fetal miscarriage (Zhang et al., 2018). CNVs are ubiquitous in the human genome, and although most are benign or VOUS, a considerable number of CNVs are associated with human diseases (Yang X. et al., 2018; Hu et al., 2018).

At present, karyotype analysis, chromosomal microarray analysis (CMA) and CNV-seq were the main methods for detecting chromosomal abnormality (Li Y. et al., 2018). Karyotype analysis is the recognized gold standard for detecting chromosomal abnormality, which can detect the numerical abnormalities and large structural abnormalities of chromosomes. Karyotype analysis requires tissue cell culture and long detection time. The technical requirements for operators are high and the stability is poor. Karyotype analysis has limited resolution and cannot detect CNV. These disadvantages limit its application (Martinez-Portilla et al., 2019). CMA is a fast and effective chromosome analysis technique, which can detect non-equilibrium chromosomal abnormalities in the whole genome through a single experiment. However, its disadvantages are low throughput and high detection cost. In addition, due to its dependence on probe hybridization, variations in areas not covered by the probe and the exact breakpoints cannot be detected (Levy and Wapner, 2018). CNV-seq is a genomic CNV detection technology based on low-depth whole-genome high-throughput sequencing technology (Zhao and Fu, 2019). CNV-seq has several advantages, including low specimen quality requirements, short detection time, high throughput, the ability to detect 100 Kb chromosomal CNV and more accurate detection of breaking points (Ellingford et al., 2017). CNV-seq can achieve the detection efficiency of CMA when CNV-seq with 1×depth (Zhou et al., 2018). Compared with other technologies based on next generation sequencing (NGS), CNV-seq test has a lower cost and can be used as a first-line prenatal diagnosis technology. In addition, a large number of CNVs found by low coverage CNV-seq have undergone secondary verification. It has been gradually applied to the detection of chromosomes in abortive tissues (Shi et al., 2019).

In this study, 505 fetal aborted tissues were examined by CNV-seq. The chromosomal abnormality rate was 54.3%, and the normal rate was 45.7%. The results are similar to those of other studies (Dai et al., 2019; Sheng et al., 2021). A total of 202 cases with numerical chromosomal abnormality, and the trisomy variation mainly occurred on chromosomes 16, 22, 21, 15, 13, and 9. The results in this study are similar to those of other studies (Liu et al., 2015; Wang M.-z. et al., 2017; Li F. X. et al., 2020). Trisomy 16 is one of the common genetic causes of early miscarriage, accounting for about 6% of early miscarriages (Martin et al., 2004). Some genes on chromosome 16 were associated with abnormal fetal head circumference (Pasternak et al., 2020), and CNVs on chromosome 16 have relationship with prenatal growth retardation (Redaelli et al., 2019). In this study, numerical chromosomal abnormalities were not detected on chromosomes 1, 11 and 19. Several studies have found that numerical abnormalities of chromosomes 1 and 19 were associated with miscarriage (Banzai et al., 2004; Vicić et al., 2008; Li H. et al., 2018). A study using CMA to detect chromosomal abnormalities in products of conception (POC) showed that the rate of abnormalities in POC specimens was 44.6%, with the most common were aneuploidies, including trisomy 16, triploidy, monosomy X, trisomy 22, trisomy 21 and trisomy 15, while the least encountered aneuploidies were trisomy one and trisomy 19 (Wang et al., 2014). The results showed that CMA and CNV-seq were similar in the detection of numerical chromosomal abnormalities in pregnancy products. In Wang BT's study, the proportion of triploidy was high (14%), while this study did not detect triploidy. In addition, one fetus with chromosome 7 tetrasomy (48,XN,+7,+7) was found in this study. Study has shown that partial tetrasomy of chromosome 7 can cause dysmorphic signs, congenital heart defect, and developmental delay (von Beust et al., 2005).

Structural chromosomal abnormality is also one of genetic factors in miscarriage except numerical chromosomal abnormality. Because the number of CNVs in the genome is so large and contains a large number of genes, identifying specific genes associated with miscarriage is a challenge. In this study, VOUS CNVs were detected in 48 fetal tissues, pCNVs and likely pCNVs were detected in 18 fetal tissues. 168 genes are involved in the VOUS CNVs and pCNVs. 41 terms and 12 pathways were enriched by GO analysis and KEGG pathway analysis in miscarriages during early and middle pregnancy, respectively. Some biological processes were enriched such as organ differentiation and development, nervous system formation and development, transmembrane transport, and cellular functions. KEGG analysis results showed that the most enriched pathways of these genes were adherens junction, amyotrophic lateral sclerosis, tight junction, and so on. The functional pathways, such as “Adherens junction” and “Tight junction” pathways may control monolayer barrier function and may be paralleled by altered cytoskeletal organization. These pathways can control barrier function by altered cytoskeletal organization (Cvitic et al., 2020). Study has also shown that “Adherens junction” was associated with repeated implantation failure (Bastu et al., 2019). ‘Tight junction’ pathway was associated with fetal neural tube defects (Wang L. et al., 2017). Amyotrophic lateral sclerosis (ALS) pathway is considered have relationship with fetuses of isolated agenesis of the corpus callosum (Chia et al., 2018; She et al., 2021). Study has shown that fetuses with isolated agenesis may be associated with amyotrophic lateral sclerosis pathway (She et al., 2021). ‘Pathways in cancer’ include Hedgehog (Hh), Wnt, phosphoinositide 3-kinase (PI3K)/protein kinase B (Akt), Janus kinase (Jak)/signal transducer and activator of transcription (STAT), mitogen-activated protein kinase (MAPK), hypoxia-inducible factor-1 (HIF-1), transforming growth factor-beta (TGF-β), vascular endothelial-derived growth factor (VEGF), and peroxisome proliferator-activated receptor (PPAR) pathways. The hedgehog pathway in the uterine stroma causes deferred implantation and embryonic loss (Harman et al., 2011). Wnt signaling pathway plays an important role in embryonic development by regulating cell differentiation, proliferation and apoptosis (Tepekoy et al., 2015; Nayeem et al., 2016). The Treg/Th17 balance serves a vital role in maintaining the steady state of the maternal-fetal interface (Qian et al., 2018). The differentiation of Treg and Th17 cells is controlled by PI3K/Akt signaling pathway (Yang Y. et al., 2018). In the early pregnancy decidua, the immunoregulation system must work to prevent fetus rejection. NK cells play very important roles in the maintenance of pregnancy (Saito et al., 2007). The JAK-STAT pathway may involve in the regulation of NK function, and it may contribute to the maintenance of immune tolerance at the maternal-fetal interface (Fu et al., 2017). Early oxygen exposure can cause oxidative damage leading to pregnancy disorders. The source of NADPH oxidase in early pregnancy may be related to the activation of MAPK pathway (Hernandez et al., 2019). Study has showed that HIF-1α/VEGF pathway may regulate villous angiogenesis in early pregnancy and HIF-1α/VEGF may be a novel biomarker for missed abortion (Zhi et al., 2018). The TGF-β signaling has been shown to regulate cell growth, immune response, and inflammation. Moreover, TGF-β is able to induce immature lymphocytes to maintain the homeostasis of the immune system. Thus, TGF-β signaling may play a role during pregnancy by regulating immune system homeostasis (Li X. et al., 2020). The peroxisome proliferator-activated receptors (PPARs) are nuclear receptors that contribute to the developmental plasticity of the placenta by regulating lipid and glucose metabolism pathways, and placental signaling pathways (Lendvai et al., 2016). Moreover, calcium signaling pathway and leukocyte transendothelial migration pathway were associated with promoting endothelial cell contraction and increased permeability, mediating inflammatory responses (Dalal et al., 2020). If there is a disorder of fetal inflammatory response, it may lead to fetal multiple organ dysfunction, and even fetal death (Jung et al., 2020).

The rate of numerical chromosomal abnormalities in fetuses of ≥35 year-old age pregnant women was higher than that in fetuses of <35 year-old age pregnant women, while the rate of structural chromosomal abnormalities in fetuses of ≥35 year-old age pregnant women was lower than that in fetuses of <35 year-old age pregnant women. It is known that reduced fertility in women over 35 years of age because maternal age is associated with the quality of oocytes. This is due to the rate of chromosome segregation errors during meiotic divisions are increasing with maternal age, and lead to the production of numerical chromosomal abnormalities (Mikwar et al., 2020). A study showed that the frequency of structural chromosomal abnormality seemed to be independent of maternal age (Xanthopoulou et al., 2012). We may need more cases to study this. The rate of chromosomal abnormalities of fetuses in early pregnancy was higher than that of fetuses in middle pregnancy. Chromosomal abnormality is the main causes of early miscarriage (Qu et al., 2019).

In addition to the above CNV detection methods, there is a new method called Optical Genome Mapping (OGM). OGM is considered as a disruptive, novel, and high resolution genome analysis technology. It has been suggested that it can be used to detect all types of genomic variations in many diseases (Pastor et al., 2020). In reproductive medicine and prenatal diagnosis, clinically significant structural variations can be detected in a single analysis by OGM. One study showed that Bianano optical mapping (BOM) can determine the number of D4Z4 repeats and exclude interference of the 10q26.3 homologous region, and in combination with karyomapping, can be used for rapid and accurate prenatal diagnosis of FSHD1 (Zheng et al., 2020). The single-molecule optical mapping (SMOM) has potential clinical application as a rapid tool to screen patients with balanced reciprocal translocations (BRTs) for underlying genetic causes of infertility and other diseases (Wang H. et al., 2020). OGM is a method that can compensate for the limitations of karyotype analysis, fluorescence *in situ* hybridization (FISH), and CMA by detecting all types of structural variations, including CNVs (Sahajpal et al., 2021).

There were some limitations in this study. First, CNV-seq technology has obvious advantages in detecting chromosomal abnormalities, but it is unable to detect chromosome structural rearrangements such as translocation, inversion, and loss of heterozygosity (LOH) such as uniparental disomy (UPD). Second, the sample size was not large enough to detect all CNVs in miscarriages during early and middle pregnancy. Third, the enrichment analysis of gene function conducted in this study was not in-depth enough. The detected genes were enriched in some functions and signal pathways, but this is just part of the functions of genes in the human body that are associated with miscarriages during early and middle pregnancy. Therefore, we need to analyze larger cohorts to screen out candidate genes related to miscarriage, and conduct basic experiments to study the mechanism of this phenomenon.

## Conclusion

The results of this study showed that CNVs were one of the genetic factors of miscarriage during early and middle pregnancy. Some meaningful genetic etiology information such as genes and pathways has been obtained, it may provide useful genetic guidance for pregnancy and prenatal diagnosis. It may provide valuable reference data for the prevention, diagnosis and treatment of miscarriage.

## Data Availability

The original contributions presented in the study are included in the article/Supplementary Material, further inquiries can be directed to the corresponding author.

## Supplementary Material

Supplementary Table 1 Summarized results of VOUS CNVs and pCNVs.
