# Supplementary material for: Analysis of Genomic Copy Number Variation in Miscarriages During Early and Middle Pregnancy
Source: Front Genet. 2021 Sep 17;12:732419. doi: 10.3389/fgene.2021.732419 (PMC8484914; doi:10.3389/fgene.2021.732419)
Supplement: Supplementary file 1 [file Table1.docx]

Supplemental Table 1 Summarized results of VOUS CNVs and pCNVs.

| Case | Maternal Age(years) | Weeks of Gestation | CNV-seq (hg19 chr: location) | Size, Mb | Clinical significance | Gene/Genes | Syndrome |
| --- | --- | --- | --- | --- | --- | --- | --- |
| 1 | 31 | 26^+4^ | dup(14)(q12)  chr4: 25700000-25860000dup | 0.16 | - VOUS | - *-* | - - |
| 2 | 37 | 9 | dup(6)(p24.1)  chr6: 13180000-13700000dup | 0.52 | - VOUS | *TBC1D7* | - - |
|  |  |  | del(8)(p11.22)  chr8: 39220000-39380000del | 0.16 | - VOUS | - *-* | - - |
| 3 | 31 | 9 | dup(16)(p13.11)  chr16: 15920000-16280000dup | 0.36 | - VOUS | *MYH11, ABCC6* | - - |
| 4 | 30 | 8^+3^ | dup(9)(p24.3)  chr9: 200000-460000dup | 0.26 | - VOUS | *DOCK8* | - - |
| 5 | 26 | 7 | dup(8)(q24.11)  chr8: 118240000-118900000dup | 0.66 | - VOUS | *EXT1, MED30* | - - |
| 6 | 24 | 8 | del(5)(q32)  chr5: 147400000-147740000del | 0.34 | - VOUS | *SPINK5, SPINK14, SPINK6, SPINK13, SPINK7, SPINK9* | - - |
| 7 | 24 | 11^+2^ | dup(X)(p22.31)  chrX: 6560000-7060000dup | 0.50 | - VOUS | *HDHD1* | - - |
| 8 | 26 | 6 | dup(4)(q22.3)  chr4: 96280000-96940000dup | 0.66 | - VOUS | *UNC5C, PDHA2* | - - |
| 9 | 29 | 9 | dup(14)(q32.33)  chr14: 104260000-104620000dup | 0.36 | - VOUS | *KIF26A, ASPG, TDRD9, RD3L, C14orf2, PPP1R13B* | - - |
| 10 | 29 | 6 | dup(8)(p22)  chr8: 15740000-16980000dup | 1.24 | - VOUS | *MSR1, FGF20* | - - |
| 11 | 35 | 16^+3^ | dup(5)(q21.1)  chr5: 98240000-98620000dup | 0.38 | - VOUS | *CHD1* | - - |
| 12 | 30 | 9 | dup(7)(p22.2)  chr7: 3020000-3320000dup | 0.30 | - VOUS | *CARD11* | - - |
| 13 | 30 | 8 | del(7)(q31.1)  chr7: 110880000-111340000del | 0.46 | - VOUS | *IMMP2L* | - - |
|  |  |  | dup(X)(p22.31)  chrX: 6540000-7060000dup | 0.52 | - VOUS | *HDHD1* | - - |
| 14 | 30 | 5 | dup(15)(q13.3)  chr15: 31960000-32420000dup | 0.46 | - VOUS | *OTUD7A, CHRNA7* | - - |
|  |  |  | dup(21)(q11.2)  chr21: 1460000-1560000dup | 1.00 | - VOUS | *LIPI* | - - |
| 15 | 32 | 13 | del(5)(q32)  chr5: 147420000-147740000del | 0.32 | - VOUS | *SPINK5* | - - |
| 16 | 30 | 9^+3^ | dup(X)(p22.31)  chrX: 650000-8120000dup | 1.62 | - VOUS | *STS* | - - |
| 17 | 40 | 6 | dup(2)(p12)  chr2: 78660000-80000000dup | 1.34 | - VOUS | *CTNNA2* | - - |
| 18 | 28 | 14 | dup(X)(p22.31)  chrX: 6540000-8120000dup | 1.58 | - VOUS | *STS* | - - |
| 19 | 28 | 7 | del(10)(q21.1)  chr10: 57080000-57720000del | 0.64 | - VOUS | *PCDH15* | - - |
|  |  |  | dup(X)(p22.31)  chrX: 6480000-8120000dup | 1.64 | - VOUS | - *STS* | - - |
| 20 | 24 | 7^+6^ | del(11)(q14.1)  chr11: 81400000-81740000del | 0.34 | - VOUS | *SLITRK6* | - - |
|  |  |  | dup(13)(q31.1q31.2)  chr13: 84480000-88120000dup | 3.64 | - VOUS | - *-* | - - |
| 21 | 30 | 7 | del(8)(p22)  chr8: 15860000-16300000del | 0.44 | - VOUS | *MSR1* | - - |
| 22 | 30 | 10 | dup(9)(q31.3)  chr9: 113080000-113660000dup | 0.58 | - VOUS | *MUSK* | - - |
| 23 | 31 | 5 | del(8)(p23.2)  chr8: 5680000-6120000del | 0.44 | - VOUS | - *-* | - - |
| 24 | 31 | 7 | dup(9)(p24.3)  chr9: 30000-660000dup | 0.36 | - VOUS | *DOCK8, KANK1* | - - |
| 25 | 28 | 7 | dup(7)(p21.1)  chr7: 17160000-17660000dup | 0.50 | - VOUS | *AHR* | - - |
| 26 | 24 | 11 | dup(X)(q28)  chrX: 152720000-152960000dup | 0.24 | - VOUS | *BGN, ATP2B3, FAM58A, SLC6A8* | - - |
| 27 | 27 | 12^+6^ | dup(22)(q11.22)  chr22: 22320000-22560000dup | 0.24 | - VOUS | *TOP3B* | - - |
| 28 | 26 | 15 | dup(3)(q26.33)  chr3: 180020000-180480000dup | 0.46 | - VOUS | *RNU6-486P, CCDC39-AS1, TTC14, RN7SL229P, CCDC39* | - - |
|  |  |  | dup(8)(q24.11)  118220000-118860000dup | 0.64 | - VOUS | *MED30, RPS10P16, EXT1* | - - |
| 29 | 39 | 6 | dup(1)(q24.3)  chr1: 171700000-171900000dup | 0.20 | - VOUS | *METTL13* | - - |
| 30 | 28 | 13^+1^ | del(11)(q14.1)  chr11: 81240000-82380000del | 1.14 | - VOUS | *MIR4300* | - - |
| 31 | 28 | 8 | dup(1)(q25.2)  chr1: 178960000-179840000dup | 0.88 | - VOUS | *NPHS2* | - - |
| 32 | 30 | 8 | dup(2)(p12)  chr2: 78620000-79980000dup | 1.36 | - VOUS | *CTNNA2* | - - |
| 33 | 36 | 9^+4^ | dup(21)(q21.1)  chr21: 19180000-19820000dup | 0.64 | - VOUS | *TMPRSS15* | - - |
| 34 | 31 | 9^+2^ | dup(7)(q22.1)  chr7: 102440000-102940000dup | 0.50 | - VOUS | *PMPCB* | - - |
| 35 | 23 | 12 | dup(X)(q13.2)  chrX: 73420000-73780000dup | 0.36 | - VOUS | *SLC16A2,* | - - |
|  |  |  | dup(X)(q21.1)  chrX: 77160000-77500000dup | 0.34 | - VOUS | *COX7B, ATP7A, PGK1* | - - |
| 36 | 34 | 9^+5^ | del(20)(p12.1)  chr20: 14700000-14980000del | 0.28 | - VOUS | *MACROD2* | - - |
| 37 | 30 | 5 | dup(7)(p22.2)  chr7: 2940000-3300000dup | 0.36 | - VOUS | *CARD11* | - - |
|  |  |  | dup(10)(q26.11)  chr10: 120540000-121300000dup | 0.76 | - VOUS | *NANOS1, SFXN4* | - - |
| 38 | 34 | 12^+2^ | dup(15)(q25.2q25.3)  chr15: 85100000-85800000dup | 0.70 | - VOUS | *WDR73, ALPK3, SLC28A1* | - - |
| 39 | 24 | 6 | dup(1)(p22.2)  chr1: 89060000-89360000dup | 0.30 | - VOUS | *CC2D2A* | - - |
|  |  |  | dup(4)(p15.32)  chr4: 15300000-15620000dup | 0.32 | - VOUS | - *-* | - - |
| 40 | 30 | 7 | dup(7)(q11.21)  chr7: 63320000-63960000dup | 0.64 | - VOUS | - *-* | - - |
| 41 | 22 | 27 | dup(9)(p24.1)  chr9: 6600000-7100000dup | 0.50 | - VOUS | *GLDC* | - - |
| 42 | 34 | 9 | dup(16)(q22.1)  chr16: 68660000-68940000dup | 0.28 | - VOUS | *CDH3, CDH1* | - - |
| 43 | 33 | 10^+1^ | dup(3)(p12.3)  chr3: 75160000-76100000dup | 0.94 | - VOUS | *ROBO2* | - - |
| 44 | 27 | 7 | dup(X)(p22.31)  chrX: 8440000-8660000dup | 0.22 | - VOUS | *KAL1* | - - |
| 45 | 24 | 8 | dup(X)(q13.3q21.1)  76000000-77100000dup | 1.10 | - VOUS | *CORIN, CNGA1, FGF16, ATRX, MAGT1* | - - |
| 46 | 27 | 18 | dup(14)(q12)  chr14: 25260000-25360000dup | 0.10 | - VOUS | - *-* | - - |
| 47 | 27 | 8 | dup(16)(p13.11)  chr16: 15500000-16300000dup | 0.80 | - VOUS | - *-* | - - |
| 48 | 28 | 14^+2^ | dup(4)(q26q27)  chr4: 200000-460000dup | 1.62 | - VOUS | *MYOZ2, SYNPO2, USP53, FABP2* | - - |
| 49 | 29 | 10 | dup(2)(p24.3)  chr2: 13520000-14320000dup | 0.80 | - Pathogenic | - *-* |  |
| 50 | 28 | 8 | dup(8)(pter-p21.1)  chr8: 100000-28660000dup | 28.56 | - Pathogenic | *MIR17HG, SLITRK6,* |  |
|  |  |  | dup(8)(q24.23-qter)  chr8: 139820000-146360000dup | 6.54 | - Pathogenic | *PUF60, TRAPPC9* | - Veiheij syndrome (OMIM 615583); Type 13 mental retardation (OMIM 613192) |
|  |  |  | dup(13)(q13.3-q14.11)  chr13: 36180000-44000000dup | 25.06 | - Pathogenic | *MADH9* | Primary pulmonary hypertension type 2 (OMIM 615342); Feingold syndrome 2 (OMIM 614326) |
|  |  |  | dup(13)(q34)  chr13: 110640000-115160000dup | 4.52 | - Pathogenic | *COL4A2* | Brain puncture malformation 2 (OMIM 614483); Predisposition to intracranial hemorrhage (OMIM 614519) |
| 51 | 28 | 7 | dup(1)(q41qter)  chr1: 223000000-249200000dup | 26.20 | - Pathogenic |  | 1q trisomy syndrome |
|  |  |  | dup(21)(q11.2q22.2)  chr21: 14600000-39960000dup | 15.36 | - Pathogenic | *CFAP298, CLDN14, SYNJ1, DONSON, HLCS, IFNAR2, IFNGR2, IL10RB, JAM2, KCNE1, TMPRSS15, TMPRSS15, PIGP, KCNE2, KCNJ6, MRAP, NRIP1, RUNX1, SOD1, SON, APP, DYRK1A* | Familial Alzheimer's disease (OMIM 104300); ZTTK syndrome (OMIM 617140); Microcephaly syndrome (OMIM 251230); Keppen-Lubinsky syndrome (OMIM 614098); 21q22.13 duplication syndrome [PMID: 27106104]; 21q22.13-q22.2 duplication syndrome [PMID: 21686961] |
| 52 | 26 | 7 | dup(7)(p22.3p21.3)  chr7: 2000000-13060000dup | 11.06 | - Pathogenic | *ACTB, AIMP2, AP5Z1, BRAT1, EIF2AK1, IQCE, KDELR2, LFNG, MRM2, PMS2, RAC1, RNF216, TMEM106B, WIPI2* | 14 pathogenic genes in OMIM |
|  |  |  | dup(7)(p21.3p14.3)  chr7: 13080000-31900000dup | 18.82 | - Pathogenic | *AHR, AQP1, CRPPA, DNAH11, FAM126A, SNX10, FKBP14, GHRHR, GPNMB, HOXA1,HOXA13, HOXA2, IL6, KLHL7, PDE1C, PPP1R17, TWIST1, CYCS, GARS1, GSDME, HNRNPA2B1, HOXA11* | 22 pathogenic genes in OMIM |
|  |  |  | del(7)(p22.3)  chr7: 60000-1920000del | 1.86 | - VOUS |  | Raine syndrome (OMIM 259775); Primary ciliary dyspnosia 18 (OMIM 614874); Prostate cancer (OMIM 176807) |
|  |  |  | dup(10)(p11.21)  chr10: 34620000-35360000dup | 0.74 | - VOUS | *PARD3, CUL2* | *-* |
| 53 | 32 | 10^+2^ | del(7)(q31.1qter)  chr7: 109000000-159120000del | 50.12 | - Pathogenic |  | 7q36 distal deletion syndrome |
|  |  |  | dup(10)(p11.21)  chr10: 34680000-35320000dup | 0.64 | - VOUS | - *PARD3, CUL2* | - - |
| 54 | 25 | 19 | del(8)(p23.3-p21.3)  chr8: 100000-23180000del | 23.18 | - Pathogenic | *GATA4* | - |
|  |  |  | dup(8)(q13.2-q24.3)  chr8: 69500000-146360000dup | 76.86 | - Pathogenic |  | DECIPHER [Patient：331008；nssv582792, 3395187] |
| 55 | 33 | 10 | del(4)(p16.3-p15.1)  chr4: 100000-32880000del | 32.88 | - Pathogenic | *-* | Wolf-Hirschhorn syndrome |
| 56 | 25 | 6^+5^ | del(8)(p23.3-p22)  chr8: 100000-18220000del | 18.22 | - Pathogenic | *-* | 8p23.1 deletion syndrome |
|  |  |  | del(8)(p22-p11.21)  chr8: 18220000-42880000del | 24.66 | - Pathogenic | *-* | [Patient:340539；nssv582794] |
|  |  |  | dup(8)(q11.21-q24.3)  chr8: 48760000-146360000dup | 97.60 | - Pathogenic | *-* | [Patient:331008；nssv582444,nssv579093] |
| 57 | 25 | 16 | del(15)(q11.2)  chr15: 22760000-23100000del | 0.34 | - Pathogenic | *TUBGCP2, NIPA1, NIPA2, CYFIP1* | 15q11.2 deletion syndrome (OMIM 615656); Spastic paraplegia 6 (OMIM 600363) |
|  |  |  | dup(17)(q22)  chr17: 53900000-54380000dup | 0.48 | - VOUS | *ANKFN1, PCTP* | *-* |
| 58 | 29 | 5 | del(5)(pterp14.1)  chr5: 20000-28080000del | 28.06 | - Pathogenic | *-* | cats cry syndrome (5p deletion syndrome) |
| 59 | 26 | 9^+5^ | dup(9)(q34.2qter)  chr9: 136240000-141020000dup | 4.79 | - Pathogenic | *ABCA2, ADAMTS13, ADAMTSL2, AGPAT2, TPRN, CACNA1B, CARD9, DBH, INPP5E, LHX3, MAN1B1, MRPS2, MYMK, PMPCA, SARDH, GRIN1, SOHLH1, TUBB4B, NSMF, EHMT1, COL5A1, KCNT1, NOTCH1* | - 23 pathogenic genes in OMIM |
|  |  |  | del(X)(p22.33p11.22)  chrX: 2720000-52100000del | 49.38 | - Pathogenic | *-* | Steroid sulfatase deficiency (OMIM 308100) |
| 60 | 30 | 9^+2^ | del(14)(q24.3qter)  chr14: 77500000-107280000del | 29.78 | - Pathogenic | *-* | Distal 14q chromatid syndrome (ORPHA: 96150) |
| 61 | 27 | 5 | del(7)(p12.2qter)  chr7: 49440000-159120000del | 109.68 | - Pathogenic | *-* | 7q partial deletion syndrome (ORPHA:262056) |
|  |  |  | del(13)(qter)  chr13: 72500000-115100000del | 42.60 | - Pathogenic | *-* | 13q distal chromatid syndrome (ORPHA:1590) |
| 62 | 28 | 11^+5^ | dup(4)(q32.1q35.2)  chr4: 157960000-189140000dup | 31.18 | - Pathogenic | *-* | 4q distal trisomy syndrome (ORPHA: 96096) |
|  |  |  | dup(11)(q23.3qter)  chr11: 118000000-134940000dup | 16.94 | - Pathogenic | *-* | 11q distal trisomy syndrome (ORPHA: 96103) |
| 63 | 27 | 12 | dup(19)(p13.3)  chr19: 280000-6100000dup | 5.82 | - Pathogenic | *-* | 19p13.3 microduplication syndrome (ORPHA: 447980) |
| 64 | 26 | 9 | dup(3)(q13.32-q28)  chr3: 118000000-189000000dup | 71.0 | - Pathogenic | *-* | Multiple pathogenic genes in OMIM |
|  |  |  | del(10)(q22.2-q22.3)  chr3: 72000000-85000000del | 13.0 | - Pathogenic | *-* | Multiple pathogenic genes in OMIM |
| 65 | 32 | 13 | dup(1)(p36.33-p34.1)  chr3: 780000-44400000dup | 43.62 | - Pathogenic | *-* | 1p36 distal trisomy syndrome |
| 66 | 32 | 8^+5^ | del(8)(q13.2-q13.3)  chr3: 69900000-72560000del | 2.66 | - Pathogenic | *-* | Otofaciocervical syndrome (OMIM 166780); Branchiootic syndrome (OMIM 602588); Branchiootorenal syndrome (OMIM 113650) |

VOUS, variants of unknown significance; XL, X-linked inheritance; XR, X-linked recessive inheritance; AD, Autosomal dominant inheritance; AR, Autosomal recessive inheritance.
